# Biophysical modeling and diffusion kurtosis imaging reveal microstructural alterations in normal-appearing white-matter regions of the brain in obstructive sleep apnea

**DOI:** 10.1093/sleepadvances/zpae031

**Published:** 2024-05-24

**Authors:** Zia Hashim, Mansi Gupta, Zafar Neyaz, Shivani Srivastava, Vinita Mani, Alok Nath, Ahmad Raza Khan

**Affiliations:** Department of Pulmonary Medicine, SGPGIMS, Lucknow, India; Department of Pulmonary Medicine, SGPGIMS, Lucknow, India; Department of Radio-diagnosis, SGPGIMS, Lucknow, India; Department of Pulmonary Medicine, SGPGIMS, Lucknow, India; Department of Neurology, SGPGIMS, Lucknow, India; Department of Pulmonary Medicine, SGPGIMS, Lucknow, India; Department of Advanced Spectroscopy and Imaging, CBMR, SGPGIMS Campus, Lucknow, India

**Keywords:** obstructive sleep apnea, diffusion MRI, microstructure, kurtosis, white-matter, CSF

## Abstract

**Study Objectives:**

Studies have indicated that sleep abnormalities are a strong risk factor for developing cognitive impairment, cardiomyopathies, and neurodegenerative disorders. However, neuroimaging modalities are unable to show any consistent markers in obstructive sleep apnea (OSA) patients. We hypothesized that, compared with those of the control cohort, advanced diffusion MRI metrics could show subtle microstructural alterations in the brains of patients with OSA.

**Methods:**

Sixteen newly diagnosed patients with moderate to severe OSA and 15 healthy volunteers of the same age and sex were considered healthy controls. Multishell diffusion MRI data of the brain, along with anatomical data (T1 and T2 images), were obtained on a 3T MRI system (Siemens, Germany) after a polysomnography (PSG) test for sleep abnormalities and a behavioral test battery to evaluate cognitive and executive brain functions. Diffusion MRI data were used to compute diffusion tensor imaging and diffusion kurtosis imaging (DKI) parameters along with white-matter tract integrity (WMTI) metrics for only parallel white-matter fibers.

**Results:**

OSA was diagnosed when the patient’s apnea–hypopnea index was ≥ 15. No significant changes in cognitive or executive functions were observed in the OSA cohort. DKI parameters can show significant microstructural alterations in the white-matter region, while the WMTI metric, the axonal-water-fraction (fp), reveals a significant decrease in OSA patients concerning the control cohort.

**Conclusions:**

Advanced diffusion MRI-based microstructural alterations in the white-matter region of the brain suggest that white-matter tracts are more sensitive to OSA-induced intermittent hypoxia.

**Statement of Significance**

We identified a significant decrease in the axonal water fraction in patients with OSA, compared with controls. Such biophysical parameters provide important cellular information, that could be useful for patient management of sleep disorders. Such findings have great translational potential for patient management. However, the inference from these parameters should be carefully ascertained. The current findings also support the idea that diffusion kurtosis imaging parameters are more sensitive to microstructural alterations than diffusion tensor imaging parameters are.

Sleep is an integral part of our routine, yet sleep abnormalities such as obstructive sleep apnea (OSA) are mostly underdiagnosed or suboptimal diagnoses that increase their severity and prevalence. OSA may cause intermittent hypoxemia, and persistent sleep disturbances are two other clinical characteristics of OSA [1, 2]. The clinical manifestations or correlates of OSA include, but are not limited to, cardiovascular disease, cognitive impairment, vascular damage, oxidative stress, cell proliferation, and apoptosis [3–5]. Many studies have linked OSA to cognitive impairment, but little is known about the microstructural alterations in the brain regions that impart cognitive impairment and neurodegeneration [6]. MRI studies of patients with OSA have shown structural and functional changes in the brain [7, 8]. Some of these changes are related to cognitive deterioration and autonomic dysfunction. Functional changes are considered early markers; however, microstructural alterations are complementary and robust tissue defects [9]. The neuroimaging methods used in these studies complement more traditional sleep-assessment techniques, such as polysomnography or neuropsychological tests [2, 10]. In the present study, we hypothesized that advanced diffusion MRI metrics have the potential to reveal significant microstructural alteration in the brain with respect to the age-matched control group.

Using cutting-edge MR imaging methods, including diffusion tensor imaging (DTI), functional MRI, and magnetic resonance spectroscopy, patients with sleep apnea exhibit abnormalities in both the white-matter and gray- matter regions of the brain [11–13]. An extension of DTI is diffusion kurtosis imaging (DKI), which enables the determination of both diffusion kurtosis metrics and the more widely utilized diffusion tensor metrics [14, 15].

In contrast to the DTI assumption of free diffusion, DKI probes non-Gaussian water diffusion in tissue. Therefore, DKI data fittings represent microstructural alterations more precisely due to the presence of cellular structural barriers and intracellular organelles that hinder free diffusion. DKI also uses higher and multiple b-values to compute its metrics (mean kurtosis [MK], axial kurtosis, radial kurtosis [RK], MK tensor [MKT], and kurtosis fractional anisotropy [KFA]) [16–18]. Few studies have assessed brain tissue microstructural alterations using DTI [7, 8, 11–13] and DKI in sleep apnea patients [10, 19, 20].

According to neuroimaging research, sleep apnea patients have significant microstructural alterations in gray and white-matter regions of the brain [12, 13, 21]. We used DTI metrics to segment the whole brain into gray matter, white matter, and cerebrospinal fluid (CSF) region-based analyses (Figure 1). In addition to DTI and DKI, we also used white-matter tract integrity (WMTI)-based biophysical model parameters, which are also based on DTI and DKI parameters [22, 23]. The WMTI model is a two-non-exchanging compartmental model involving the intra-axonal space and extra-axonal space in parallel aligned white matter fiber bundles. The WMTI model assumes that the water diffusion is anisotropic Gaussian in the chosen fibers. The principal parameters of this model are intra-axonal diffusion tensors and extra-axonal diffusion tensors, the axonal water fraction, and the tortuosity of the extra-axonal space. However, as branch 1 (the intra-axonal axial diffusivity (Daǁ) is greater than the extra-axonal axial diffusivity (Deǁ)) is more plausible than branch 2 (Deǁ>Daǁ), only branch 1 outputs were computed for further statistical analysis [24].

**Figure 1.**
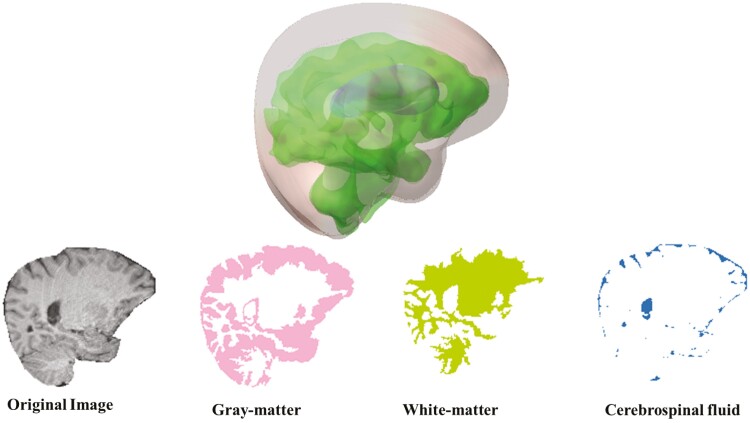
A 3D representation of the brain and segmented gray matter, white matter, and CSF from an original image.

The present study used the axonal water fraction, intra-axonal diffusivity, extra-axonal diffusivity, and extra-axonal space tortuosity. Significant alterations in OSA patients in comparison to controls were probed by DKI and WMTI metrics in the present study. These findings suggest that OSA is more sensitive to white-matter brain tissue microstructure than gray-matter. Additionally, advanced diffusion MRI metrics have shown potential to for revealing subtle microstructural alterations in the normal-appearing brains of patients with OSA. Such metrics could be used for the early diagnosis and prognosis of subtle radiological disorders [25].

## Materials and Methods

### Study design and participants

The study was approved by the Institutional Ethics Committee (Ethics No. 2019-167-IMP-111: IEC, Sanjay Gandhi Post Graduate Institute of Medical Sciences (SGPGIMS), Lucknow) of our department at a tertiary care hospital in India, comprising 16 newly diagnosed patients with moderate to severe OSA (age range 18–71 years; 14 men and 2 women) and 15 healthy volunteers of the same age and sex (14 men, 1 woman) serving as healthy controls. There was also no significant difference in the group according to the gender factor. All adult patients over the age of 18 who visited our sleep clinic OPD for the evaluation of suspected OSA were examined. Patients with an established OSA diagnosis, those receiving ongoing continuous or bilevel positive airway pressure (CPAP or BiPAP) therapy, and those exhibiting clinical signs of additional sleep disorders such as restless legs syndrome, and chronic insomnia were excluded from the study. The study also omitted patients with advanced respiratory failure requiring long-term oxygen therapy, active infections, substance abuse, neuromuscular diseases, stroke, history of heart failure, chronic kidney disease, hypothyroidism, uncontrolled diabetes, malignancies, neuropsychiatric conditions, and MRI examination contraindications such as metallic implants and claustrophobia. Patients who had structural and evident focal lesions on anatomical MRIs were also excluded. After obtaining the patient’s informed consent, only patients who met the inclusion criteria were recruited.

All the details of the patient’s symptoms and comorbidities were collected at the initiation of the study. Each participant’s medical history, vital signs, height, weight, and body mass index (BMI) were recorded. The probability of OSA was assessed using the STOP-BANG score and the Epworth Sleepiness Score (ESS) in all patients [26, 27]. The ESS score was used to ask questions about EDS. A score of 11 or higher was considered significant (0 to 5: normal lower limit, 6–10: normal and above, 11–12: slightly excessive, 13–15: moderately excessive, 16–24: severe EDS). All participants were also questioned about various parameters of the STOP-BANG questionnaire. This questionnaire included eight bifurcated items (snoring, fatigue, observed apnea, hypertension, BMI [> 35 kg/m2], age [> 50 years], neck circumference [> 43 cm for men, > 41 cm for women], and male sex), with single values of 0–2, 3–4, and 5–8 indicating low, moderate, and high risk of OSA, respectively [26, 28]. To rule out numerous comorbidities, such as uncontrolled diabetes mellitus, severe hypertension, dyslipidemia, and hypothyroidism, patients additionally underwent routine examinations. Within 2 weeks of their evaluation in the sleep clinic OPD, those patients who met the eligibility criteria were then given dates for their sleep investigations that were best suited for them.

### Polysomnography

An overnight polysomnography study and continuous/two-stage positive airway pressure (CPAP/BiPAP) titration (if required) were carried out in adherence with the most recent American Academy of Sleep Medicine (AASM) guidelines [29, 30] in our designated sleep laboratory. The following 32 channels were used to record information: sleep stage (4-channel EEG, electrooculogram, chin electromyogram, electrocardiogram channel, nasal and oral airflow (nasal thermistor and cannula), thoracic and abdominal respiratory movements (respiratory impedance), oxygen saturation (pulse oximetry), snoring (microphone), and body position, respiratory impedance, oxygen saturation, pulse oximetry, snoring, and body position). These Polysomnography (PSG) results were recorded for further sleep analysis after being manually reviewed in the laboratory by competent and seasoned sleep professionals. According to the standard AASM manual of scoring of sleep and associated events [29, 30], the stages of sleep and respiratory episodes were assessed. There were apneas and hypopneas among the abnormal respiratory events. The cessation of airflow for ≥ 10 seconds at a ≥ 90% reduction from the baseline was referred to as apnea. Hypopnea was defined as either a reduction in airflow of ≥ 30% for ≥ 10 seconds while also experiencing a ≥ 3% desaturation or arousal or a ≥ 30% drop in airflow for ≥ 10 seconds while also experiencing a ≥ 4% reduction in airflow relative to baseline. The average number of apnea and hypopnea events per hour of sleep was known as the apnea–hypopnea index (AHI). Additional events include hypopneas, obstructive apneas (airflow cessation when the respiratory effort is present), central apneas (airflow cessation when the respiratory effort is absent), and mixed apneas (when central apnea is followed by an obstructive element). OSA was diagnosed when a patient’s AHI was ≥ 15 or when a patient with symptoms suggestive of OSA had a total sleep time AHI of ≥ 5/hour [31–33].

## Neurocognitive Assessment

### Montreal cognitive assessment

The cognitive domains of individuals under visuospatial and executive functions were assessed using the montreal cognitive assessment (MOCA) scale, which included subtests such as trail making, cube copying, clock drawing, naming, attention, language, memory, and orientation to time and place [34]. The total score ranges from 0 to 30 points, where a higher score indicates superior cognition.

### Trail making test

Patients’ visual attention, praxis processing speed, and cognitive flexibility, primarily focusing on executive functions, were assessed by the trail making test (TMT) test. It consists of two parts: part A, where numbers are randomly distributed, and part B, where alternating numbers and letters are connected [35]. We evaluated individuals based on their TMT-Part A scores. Lower scores indicated better executive functions.

### MRI data acquisition:

All participants were scanned using a three Tesla MRI scanner (Siemens Magnetom Skyra, Germany) equipped with a 64-channel RF head coil. The ~30-minute protocol included conventional MRI sequences, such as T1-weighted-fluid-attenuated inversion recovery (T1 FLAIR) images with relaxation and echo times (TE/TR: 2.49/250 ms, 40 slices, 3-mm slice thickness), and T2 weighted images with TE/TR: 100/6000 ms, 2 mm slice thickness with 60 slices.

Diffusion data were obtained using a single-shot twice-refocused 2D spin-echo echo-planar imaging sequence with a TE/TR of 110/11 000 seconds, two signal averages, 30 nonlinear diffusion-weighted gradient directions, 3 diffusion weightings (b = 0, 1000, 2000 s/mm2), and an acquisition time of ~20 minutes. The matrix size of each image is 104 × 100 × 60 with an image resolution of 2.5 × 2.5 × 2.0 mm. The whole brain is covered with ~60 slices in the coronal plane, with a similar orientation as in the anatomical images.

### Image preprocessing and DTI, DKI, and WMTI metric computation

All diffusion-weighted images were preprocessed before DTI and DKI metric computation as described previously [23, 36–39]. Briefly, all diffusion-weighted images were registered to corresponding b0 images using affine registration. Denoising of diffusion MRI data was performed as described recently, followed by Rician-noise correction and Gibbs-ringing correction [40, 41]. All DTI and DKI maps were computed using robust and reproducible DKI metrics [42]. WMIT masks were created for individual brains based on different DTI-based parameters, such as linearity (cL < 0.4), planarity (Cp > 0.2), sphericity (Cs > 0.35), and radial diffusivity (RD < 0.05). The WMTI tract-based parameters were computed, viz. axonal water fraction (fp), intra axonal diffusivity (Da; µm2/ms), extra axonal diffusivity (De; µm2/ms), and tortuosity (tor) of white matter tracts of the brain.

### Statistical analysis

STATA 18.0 software (StataCorp. 2019; Stata Statistical Software: Release 18. College Station, TX: StatCorp LLC) was used for the statistical analysis. Descriptive statistics (median, interquartile range) were used to present demographic and clinical (continuous) assessment information. Data with proportions are expressed as percentages. To compare continuous variables, the Kruskal‒Wallis test was used. The normality of the data for continuous variables was tested using the Shapiro‒Wilk test. All tests were two-tailed and considered significant at the >5% level. When comparing categorical variables, Fisher’s exact test was used. Statistics were deemed to be significant at *p* < .05.

## Results

The clinical findings of the patients and controls are given in Table 1. The patients were similar to the controls in terms of age and comorbidities. There was a history of excessive daytime somnolence in patients, which was indicated by an Epworth Sleepiness Scale score of 15.0 (13.5, 16.5). Comorbidities such as hypertension, diabetes, and hypothyroidism were similar in both groups. There was no history of memory loss, cranial nerve palsy, or any focal neurological deficits. The detailed neurological examination was normal in both the patients and controls. All patients had OSA, as indicated by a median AHI of 36.3 (32.3, 39.4) ranging from 31.3 to 51.4.

**Table 1.** Clinico-Demographic Data

Cases	Controls	Test	*P*-value
*N*	16.0 (51.6%)	15.0 (48.4%)	
Age	52.0,44.0,58.5	47.0,44.0,50.0	.228
BMI	28.2,26.6,32.6	27.0,25.5,27.6	.036
ESS	15.0,13.5,16.5	5.0,2.0,8.0	<.001
STOP BANG	5.0,4.5,7.0	2.0,1.0,3.0	<.001
Hypertension	8 (50.0%)	6 (40.0%)	.722
Diabetes	6 (37.5%)	4 (26.7%)	.704
Hypothyroidism	1 (6.2%)	2 (13.3%)	.600
MOCA	27.0,26.0,28.0	28.0,26.0,29.0	.114
MMSE	25.0,23.0,27.0	27.0,26.0,28.0	.038
TMT	24.5,20.5,28.5	22.0,19.0,25.0	.185

ESS, Epworth sleepiness score; EDS, Excessive daytime somnolence.

^*^(Median, Quartile 1, Quartile 3).

Level I PSG was performed in both the patients and controls. The PSG results are shown in Table 2. Sleep efficiency and percentages of different stages were similar in both patients and controls. However, the patients had a greater apnea–hypopnea index than the controls.

**Table 2.** Polysomnographic Data

	Cases *N* = 16	Controls *N* = 15	Significance
SE	82.0(81.2,83.1)	82.6(81.4,86.0)	0.213
Latency	13.0(3.6,32.5)	12.5(2.5,73.0)	0.489
N1	13.3(12.6,14.7)	13.6(12.7,14.6)	0.828
N2	35.0(34.6,39.4)	39.3(35.1,41.3)	0.069
N3	24.2(21.3,25.8)	24.3(21.4,25.8)	0.890
REM	26.2(21.7,28.7)	23.5(20.3,25.7)	0.213
SpO2 < 90% (minutes)	34.8(21.3,46.6)	0.0(0.0,1.4)	<0.001
AHI	35.3(33.6,47.6)	1.6(1.2,2.5)	<0.001
REM-AHI	43.9(39.8,53.3)	2.0(1.5,2.8)	<0.001

SE, Sleep efficiency, AHI, apnea–hypopnea index, REM-AHI, apnea–hypopnea index during rapid eye movement sleep.

^*^(Median, Quartile 1, Quartile 3).

Neurological examination of both the patients and controls revealed grossly normal results. There was no decrease in power or sensation in any part of the body. The higher mental examination was assessed by the Mini-Mental State Examination (Folstein 1975), the MoCA test, and the TMT. There was no significant difference observed in any of the higher mental function tests.

**Figure 2.**
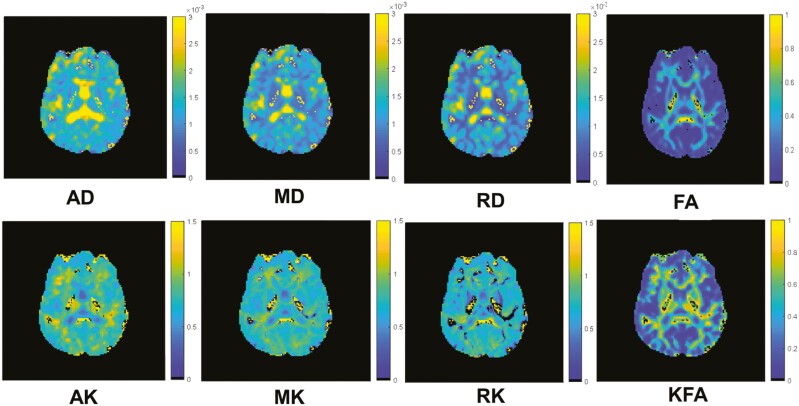
First-row diffusion tensor imaging maps: Axial Diffusivity (AD), Mean Diffusivity (MD), Radial Diffusivity (RD), and Fractional Anisotropy (FA) and Second-row diffusion kurtosis imaging maps:. Axial Kurtosis (AK), Mean Kurtosis (MK), Radial Kurtosis (RK), and Kurtosis Fractional Anisotropy (KFA).

No brain pathology or abnormalities were observed in the anatomical brain scans. A brain slice with DTI maps in the first row and DKI maps in the second row is shown in Figure 2. Very few voxels (<1%) of the whole image usually show extreme values at the cerebrospinal fluid and tissue interface and around.

### DTI metrics

**Figure 3.**
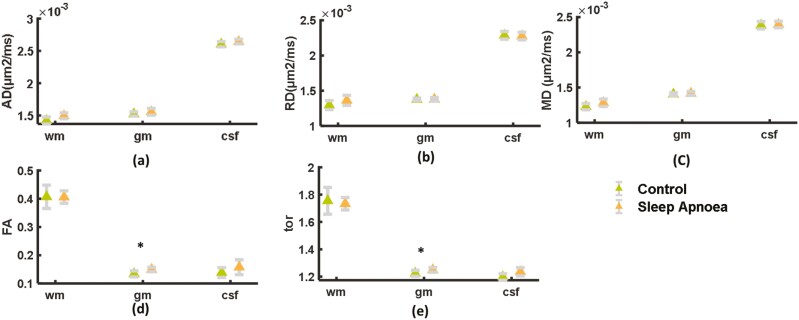
Diffusion tensor imaging parameters in white-matter (wm), gray-matter (gm), and cerebrospinal fluid (CSF) regions of the brain. Axial Diffusivity(AD) (µm^2^/ms), Radial Diffusivity (RD; µm^2^/ms), Mean Diffusivity (MD; µm^2^/ms), FA (Fractional Anisotropy), and tortuosity (tor: AD/RD) parameters. Significant alterations are represented by * (*p* < .05).

Among the DTI metrics FA showed a significant increase, in the gray matter region of the brain (*p* < .05; Figure 3D) in comparison to the control. Tortuosity (AD/RD) also showed a significant increase in the gray matter region in comparison to the control (*p* < .05; Figure 3E). No other DTI metrics showed any significant or marked alterations in the OSA patients in comparison to the controls.

### DKI metrics

**Figure 4.**
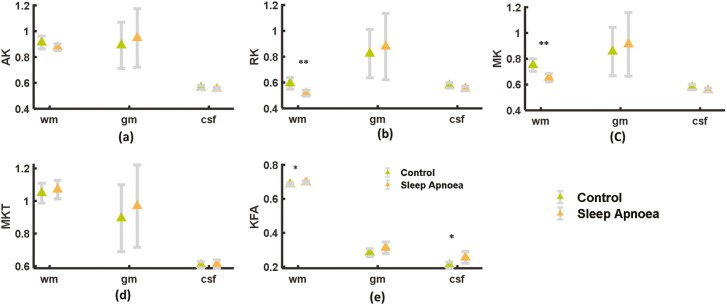
Diffusion kurtosis imaging parameters in white-matter, gray-matter, and cerebrospinal fluid (CSF) regions of the brain. Axial Kurtosis (AK), Mean Kurtosis (MK), Radial Kurtosis (RK), and KFA (Kurtosis Fractional Anisotropy) parameters. Significant alterations are represented by * (*p* < .05), ** (*p* < .01).

Contrary to DTI metrics, DKI metrics have shown significant alterations in the white matter region of the brain of patients with OSA in comparison to controls. A significant decrease in RK (*p* < .01; Figure 4B) and MK (*p* < .01; Figure 4C) was observed only in the white matter region of the brain in comparison to the control, while KFA showed a significant increase in the white matter region of the brain and cerebrospinal fluid region (*p* < .05; Figure 4E) in comparison to the control.

### WMTI metrics

**Figure 5.**
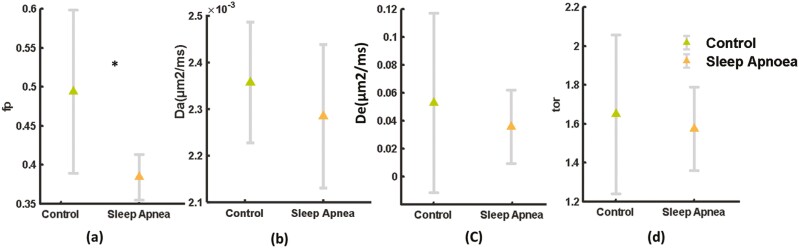
White-matter tract integrity parameters: axonal water fraction (fp), Intra axonal diffusivity (Da) in (µm^2^/ms), Extra axonal diffusivity (De; µm^2^/ms), and Tortuosity (tor) of white matter tracts of the brain. Fp is significantly reduced (*p < .05) in obstructive sleep apnea, while Da, De, and tor have not shown any significant alteration.

Among the WMTI metrics, only fp was significantly lower (*p* < .05) in the OSA group than in the control group (Figure 5A). However, Da was lower in the OSA group than in the control group, but the difference was not significant (Figure 5B). De and tortuosity did not change significantly.

**Figure 6.**
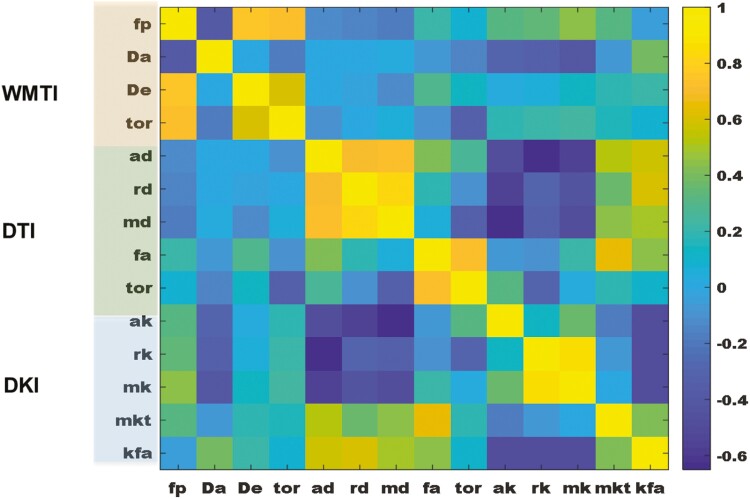
A correlation heat map of all the white-matter tract integrity tract metrics (fp, Da, De, and tor), diffusion tensor imaging metrics (ad, rd, md, fa, and tor(ad/rd), and diffusion kurtosis imaging metrices (ak,rk,mk,mkt, and kfa).

A correlation analysis of the WMTI metrics, DTI, and DKI metrics was performed to explore how WMTI metrics (biophysical parameters with specific histological information) can provide a better understanding of the DTI and DKI metrics of corresponding voxels. Significant correlations were detected between fp and MK (*p* < .05 and *r* = .45), Da and mk (*p* < .05, *r* = .42), and Da and KFA (*p* < .05, *r* = .39) Figure 6.

## Discussion

This study showed that DTI metrics show weak microstructural alterations. The DTI metrics show only significant alterations in the gray matter and CSF regions employing FA and tortuosity parameters, respectively. An increase in CSF tortuosity may reflect sleep deprivation and inefficient glymphatic waste clearance of the brain parenchyma due to reduced CSF-interstitial fluid (ISF) movement and the accumulation of CSF biomarkers and inflammatory cells in the CSF [6, 13]. Abnormal sleep patterns may lead to the accumulation of proteins and metabolites in CSF, which is likely to increase the tortuosity of CSF [43]. An insignificant rise in the FA of CSF and a significant increase in the KFA of CSF also support the speculation of abnormal waste clearance of the brain parenchyma. Although FA and KFA are sensitive measures of the microstructural complexity of the tissue, these parameters are less specific to whether the change occurred in the axial, radial, or both directions of the axons. The significant increase in gray-matter FA is contrary to the findings of some previous studies, where a significant decrease in FA was observed in gray and white-matter regions of the brain in OSA patients in comparison to controls [9, 44, 45].

The present study did not observe any significant alterations in the axial diffusivity (AD) or radial diffusivity (RD) parameters of DTI, while a previous study revealed a significant reduction in AD and RD in the brains of patients with OSA and inferred these findings as abnormal myelin and axonal integrity [11]. However, an increase in FA in the gray-matter region could be due to cerebral edema, and astrogliosis, which are features of neuroinflammation [46, 47] that have also been reported in OSA patients [7]. However, the association of FA with neuroinflammation has not yet been validated, but few studies have validated FA with 3D microscopic data [48, 49].

Additionally, the MK and MKT of the gray matter region did not increase significantly in the present study. MK has previously been shown to be significantly positively correlated with neuroinflammation [50]. In comparison to DKI parameters, DTI parameters do not reflect robust microstructural alterations, while DKI parameters have shown a significant alteration in the white-matter region according to RK, MK, and KFA in patients with OSA in comparison to controls. A previous study also showed a significant decrease in global MK in SA patients in comparison to controls [20]. Reduced kurtosis values may reflect tissue atrophy, particularly in white-matter regions of the brain, in comparison to control, which also strengthens the notion that white-matter is more sensitive to sleep disturbances [12].

A recent study utilizing DTI, DKI, and the spherical mean technique (a biophysical model) on acute sleep deprivation also showed that even 24 hours of sleep deprivation causes significant white-matter alterations in the brain [19]. The study further emphasized that 32 hours of sleep deprivation causes significant extra-axonal white matter to appear more sensitive to sleep deprivation. However, contrary to these findings, our biophysical model parameters based on WMTI-tract metrics revealed a significant reduction in the fp and a marked decrease in intra-axonal diffusivity (Da) in patients with OSA in comparison to controls.

A significant positive correlation between fp and MK also reflects that a decrease in axonal microstructure likely leads to a decrease in MK. Such correlations provide a plausible interpretation of clinically relevant DKI metrics. Otherwise, a plausible interpretation of DTI and DKI metrics is difficult to understand in terms of cellular alterations. Therefore, the biophysical model parameters of diffusion MRI data are potentially very useful for interpreting microstructural alterations in normal-appearing white-matter structures. Although most of the biophysical model parameters require a larger diffusion-weighted image dataset with higher b-values, WMTI-tract parameters can be computed with a clinically relevant diffusion MRI dataset. Despite having advantages, biophysical model parameters have limitations, such as WMTI-tract metrics that can be computed only for parallel white-matter fibers [22]. Therefore, it represents only a fraction of the total white-matter structure. A reduction in the axonal water fraction not only infers axonal injury but also may indicate demyelination of axons. A marked decrease in intra-axonal diffusivity also supports this plausible interpretation. As we did not observe any robust significant alterations in DTI metrics, widespread axotomy in the white-matter region of the brain seems unlikely, although neuronal cell apoptosis has been observed in a mouse model of chronic intermittent hypoxia [12].

OSA-induced intermittent hypoxia might alter cellular osmoregulation in the brain [51], and could be a plausible reason for a significant reduction in fp and a marked decrease in Da in the white-matter region of the brain with respect to the control. Nevertheless, advancements in biophysical modeling parameters based on advanced artificial intelligence may pave the way to computing parameters so that diffusion MRI can be used as a virtual microscope [52].

The small cohort of control and patients with OSA is the foremost limitation of the study. The OSA cohort also did not show any significant behavioral or neurological alterations. Additionally, polysomnography study-based parameters, viz., SE and latency, were also not significantly altered in the patients with OSA compared with controls, possibly due to the small cohort size and/or variable severity of OSA.

## Conclusion

This cross-sectional study in patients with OSA demonstrated significant abnormalities in various DKI parameters using whole-brain analysis, where gray matter, white matter, and CSF were segmented to study overall microstructural alterations in the brains of patients with OSA. As several gray-matter and white-matter regions often reflect contrasting outcomes, the overall effect may be helpful for a better understanding of microstructural alterations in the brain. The present study also showed that DKI metrics captured more subtle tissue structural abnormalities than DTI metrics. This also supports that, DKI metrics are more sensitive in demonstrating abnormalities in tissue structural organization at the microstructural level before any detectable changes appear in anatomical MR images. In addition to DKI, biophysical modeling of diffusion MRI data can provide useful information at the cellular and subcellular levels to better understand the microstructural complexity of DTI and DKI findings. These findings indicate the importance of using DKI and biophysical modeling for future in-depth studies to evaluate the brain tissue microstructural changes in patients with OSA and to determine the associations of these changes with clinical signs or symptoms and cognitive functions.

## Data Availability

The data underlying this article will be shared upon reasonable request to the authors.
